# ﻿*Gastrochilusheminii* (Orchidaceae, Epidendroideae), a new species from Sichuan, China, based on molecular and morphological data

**DOI:** 10.3897/phytokeys.215.91061

**Published:** 2022-12-16

**Authors:** Min Liao, Yue-Hong Cheng, Jun-Yi Zhang, Yu Feng, Gui-Ying Liu, Ping Ye, Sen-Long Jin, Hong-Qiang Lin, Bo Xu

**Affiliations:** 1 China-Croatia “Belt and Road” Joint Laboratory on Biodiversity and Ecosystem Services, CAS Key Laboratory of Mountain Ecological Restoration and Bioresource Utilization & Ecological Restoration and Biodiversity Conservation Key Laboratory of Sichuan Province, Chengdu Institute of Biology, Chinese Academy of Sciences, Chengdu 610041, China Chengdu Institute of Biology, Chinese Academy of Sciences Chengdu China; 2 University of Chinese Academy of Sciences, Beijing 10049, China University of Chinese Academy of Sciences Beijing China; 3 Wolong National Natural Reserve Administration Bureau, Wenchuan 623006, Sichuan, China Wolong National Natural Reserve Administration Bureau Wenchuan China; 4 College of Life Sciences, Chongqing Normal University, Chongqing 401331, China Chongqing Normal University Chongqing China

**Keywords:** *Gastrochilus*, morphological, Orchidaceae, phylogeny, taxonomy

## Abstract

*Gastrochilusheminii* (Orchidaceae), a new orchid species from Sichuan Province, Southwest China, is described and illustrated. It morphologically resembles *G.affinis* and *G.yei*, but differs markedly from the former in having a thinner and slightly rolled downwards reniform epichile and the central thickened purple-red mat with irregular folds (vs. subtriangular epichile curves upwards, with 2 thick, brown to purplish-brown median ridges from base to apex), and can be clearly distinguished from the latter by having reniform epichile with lobed apex and subconical hypochile with bilobed apex that splits into two conical protrusions (vs. semi-rounded epichile not lobed and subconical hypochile not bilobed). The results of molecular phylogenetic analysis based on nuclear ribosome internal transcribed spacer (nrITS) and four chloroplast DNA fragments (*mat*K, *psb*A-*trn*H, *psb*M-*trn*D, and *trn*L-F) of 36 *Gastrochilus* species showed that *G.heminii* was closely related to *G.affinis* and *G.distichus*.

## ﻿Introduction

*Gastrochilus* D.Don (Orchidaceae, Epidendroideae; Don 1825) is diagnosed by a subdivided labellum with a recurved epichile and a saccate hypochile attached to the column at the base and two subglobose pollinia attached to a slender and filiform stipe (Tsi 1999; Pridgeon et al. 2014). The genus comprises more than 70 accepted species, most of which are distributed in Southeast Asia (Kumar et al. 2014; Liu et al. 2016, 2019; Liu and Gao 2018; Rao et al. 2019; Govaerts et al. 2021; Nguyen et al. 2021; Zhou et al. 2021a, b; Zhang et al. 2022). Liu et al. (2019) first constructed the phylogenetic relationships of *Gastrochilus* and divided them into five clades. Recently, the phylogenetic studies by Liu et al. (2020), Li et al. (2022), and Zhang et al. (2022) also supported the monophyly of *Gastrochilus*.

An unusual arboreal miniature orchid was recently discovered for the first time in Wolong Nature Reserve (Wenchuan County, Sichuan Province, China) during a routine survey. It was tentatively identified as a species of Gastrochilussect.Microphyllae Bentham & Hooker (Bentham and Hooker 1883), characterized by plants with extremely small flowers, distichous and alternate leaves compared to plants in the other sections (larger flowers or clustered leaves). However, a morphological examination revealed that this species shows an unusual combination of characters that does not match any known species of G.sect.Microphyllae. The phylogenetic analysis using five DNA markers (nrITS, *mat*K, *psb*A-*trn*H, *psb*M-*trn*D, and *trn*L-F) confirmed the monophyly of this taxon. The objectives of this study are (1) to describe, (2) to examine both molecular and morphological affinities of this new *Gastrochilus* species, *Gastrochilusheminii* M.Liao, B.Xu & Yue.H.Cheng, sp. nov.

## ﻿Materials and methods

### ﻿Morphological analyses

The measurements and description of *Gastrochilusheminii* were based on two living plant individuals and two herbarium specimens (voucher information: *Min Liao & Yue-Hong Cheng ZJY143*; *Min Liao & Yue-Hong Cheng ZJY167*), respectively. The taxonomic description follows the terminology used by Beentje (2012). Voucher specimens and additional silica-gel dried leaves are deposited in CDBI Herbarium (herbarium follows Thiers 2021).

### ﻿DNA extraction, amplification and sequencing

The sequences of the two individuals of the new species newly generated in this study, and the sequences of the remaining 42 species used in the molecular phylogenetic analysis, were retrieved from GenBank. The information on the DNA fragments and four complete plastid genomes were listed in Appendix 1. Total DNA was extracted exclusively from silica-gel dried leaves via a Plant DNA Isolation Kit (Cat.No.DE-06111). We used the same primers as Liu et al. (2019) to amplify the nuclear ribosome internal transcribed spacer (nrITS) and the four chloroplast DNA fragments (i.e., *mat*K, *psb*A-*trn*H, *psb*M-*trn*D, and *trn*L-F) through polymerase chain reaction (PCR). All DNA samples were sent to TSINGKE Biotech Co. Ltd (Chengdu, China) for sequencing. The sequences were then deposited with GenBank, with the following accession numbers: *G.heminii*, nrITS (ON286752, ON286753), *mat*K (ON331126, ON331127), *psb*A-*trn*H (ON331128, ON331129), *psb*M-*trn*D (ON331130, ON331131), and *trn*L-F (ON331132, ON331133).

### ﻿Phylogenetic analyses

All sequences were edited via Sequencher v4.1.4 (Gene Codes, Ann Arbor, Michigan, USA) and aligned via MAFFT v7.475 (Katoh and Standley 2013) with default parameters. We performed phylogenetic analyses based on combined nuclear ribosome internal transcribed spacer (nrITS) and the four chloroplast DNA fragments. The nucleotide substitution model for the data matric was estimated using jModeltest v2.1.6 (Posada 2008) software and the best-fit model (GTR+I+G) was selected using the corrected Akaike Information Criterion (AICc). Two different methods, Maximum likelihood (ML) and Bayesian inference (BI), were employed. The ML analysis was performed using IQ-TREE v1.4.2 (Nguyen et al. 2014) with branch support estimated by 2,000 replicates of ultrafast bootstrapping algorithm (UFboot) (Minh et al. 2013). The BI analysis was conducted using MrBayes v3.2.7a (Ronquist and Huelsenbeck 2003), with two separate Markov-chain Monte Carlo (MCMC) chains (1,000,000 generations and sampled every 1,000 generations). The first 25% of the trees were discarded as burn-in, and the remaining trees were used to generate a majority-rule consensus tree.

## ﻿Results

**Figure 1.**
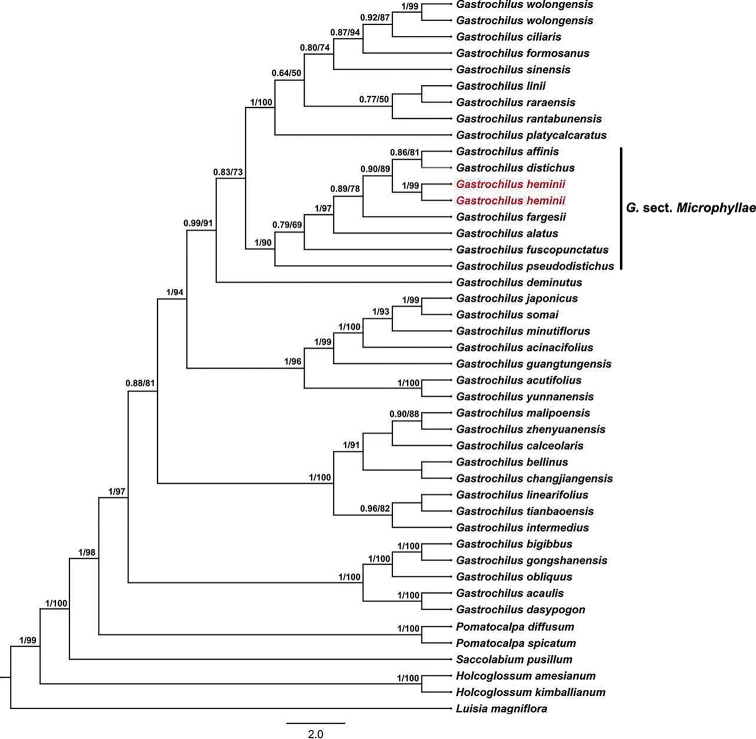
Maximum likelihood tree of *Gastrochilus* from phylogenetic analysis of combined nrITS and plastid DNA markers. Numbers before and after the slash indicate Bayesian posterior probabilities and ML bootstrap supports, respectively. The position of *Gastrochilusheminii* is indicated in red.

The molecular phylogenetic tree showed that the 36 taxa of *Gastrochilus* formed a well-supported monophyletic group (BI/ML = 1/97, Fig. 1). The two individuals of *G.heminii* were resolved as sisters to each other (BI/ML = 1/99, Fig. 1). Our data recovered a sister relationship between *G.affinis* (King & Pantl.) Schltr. (King and Pantling 1898; Schlechter 1913) and *G.distichus* (Lindl.) Kuntze (Lindley 1859; Kuntze 1891); these two species formed a monophyletic group with *G.heminii* (BI/ML = 0.90/89, Fig. 1), which formed a subclade of section Microphyllae together with *G.fargesii* (Kraenzl.) Schltr. (Kraenzlin 1903; Schlechter 1919), *G.alatus* X.H.Jin & S.C.Chen (Jin et al. 2007), *G.fuscopunctatus* (Hayata) Hayata (Hayata 1912, 1917), and *G.pseudodistichus* (King & Pantl.) Schltr. (King and Pantling 1895; Schlechter 1913).

**Figure 2.**
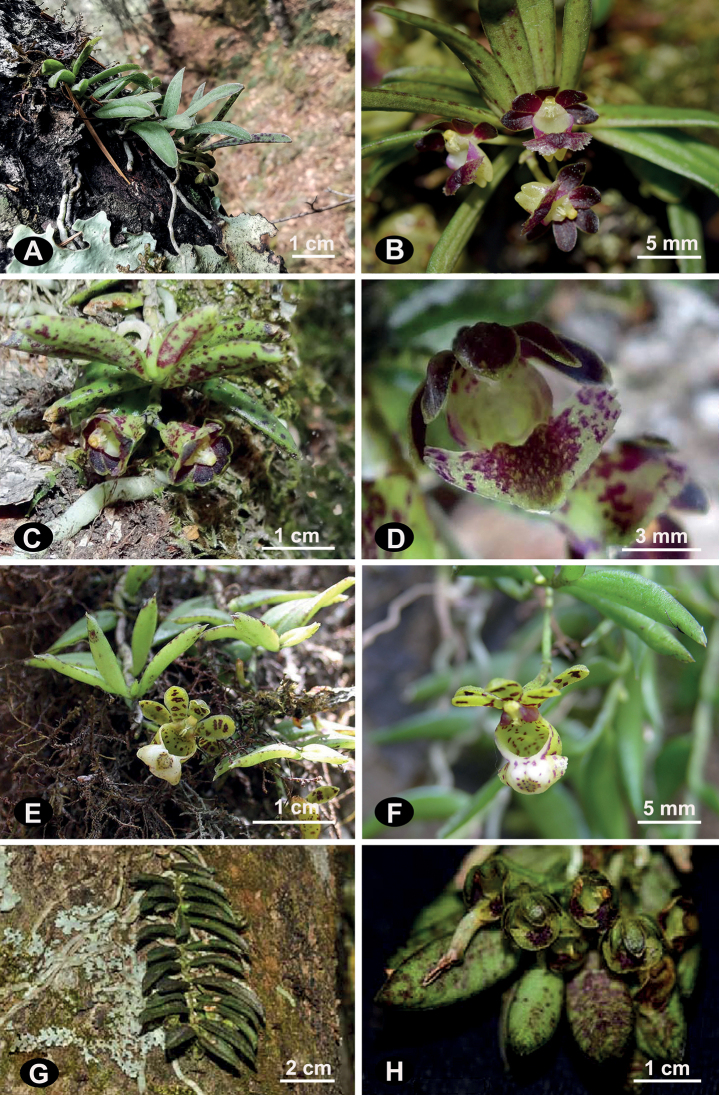
Comparison of three taxa of *Gastrochilus***A, B***Gastrochilusheminii***C, D***G.affinis***E, F***G.distichus***G, H***G.yei.* [Images **C, D** cited from Jalal et al. 2020; image **E** reproduced from website (http://www.orchidspecies.com/gastdistichus.htm); image **F** cited from Kumar et al. 2014, which was photographed by Xiao-Hua Jin; images **G** and **H** cited from Li et al. 2021].

Morphologically, *G.heminii* is closest to *G.affinis* and *G.yei* JianW.Li & X.H.Jin (Li et al. 2021). A thorough morphological comparison among *G.heminii*, *G.affinis*, *G.yei* and *G.distichus* is summarized in Table 1 and Fig. 2.

**Table 1.** Morphological comparison of *Gastrochilusheminii* with three related species, *G.affinis*, *G.distichus* and *G.yei*.

Character	*G.heminii*	*G.affinis*	*G.distichus*	*G.yei*
Plant length	3.0–6.5 cm	4.0–15.0 cm	1.5–20.0 cm	3.0–8.0 cm
Leaf shape	narrowly oblong or falcate oblong	oblong-lanceolate to subspathulate	lanceolate or falcate lanceolate	blade lanceolate
No. of flowers per inflorescence	1 or 2 flowers	1–4 flowers	2–4 flowers	2–6 flowers
Peduncle length	0.4–0.7 cm	1.5–2.0 cm	1.0–1.5 cm	0.7–1.0 cm
Dorsal sepal	elliptic-oblong, ca. 2.4 × 1.5 mm, concave, apex obtuse	elliptic-oblong, 3–5 × 1–1.3 mm, concave, apex obtuse	oblong-elliptic, 4.5–0.5 × 2.5–3 mm, concave, apex obtuse	oblong, 3.3 × 1.9–2.0 mm, apex rounded
Lateral sepals	similar to dorsal sepal, equal in size	elliptic-ovate, 3.5–4 × 0.7–1.3 mm, slightly oblique and incurved, apex obtuse	similar to dorsal sepal, equal in size	oblong, 3.9–4.0 × 1.8–1.9 mm, apex obtuse
Petals	narrowly oblong, ca. 2.6 × 1.3 mm, apex acute, base narrowed	ovate-elliptic to elliptic, 3–4 × 1–1.3 mm, apex obtuse	subobovate, slightly smaller than sepals, apex obtuse	oblong, 3.5 × 1.8 mm, apex rounded
Epichile	reniform, 4.2–6.5 × 2.0–3.0 mm, margin erose, smooth and glabrous above, central thickened purple-red mat with irregular folds	subtriangular, decurved, subacute at apex, margin finely erose at base, disk with 2 thick, brown to purplish-brown median ridges from base to apex.	subcircular, 5 × 3 mm, apex obtuse, margin entire, smooth and glabrous above and thickened cushion-like centrally, with 2 conical callosities near base	semi-rounded, 2.0–2.2 × 4.0–4.2 mm, glabrous, with a thicken central, rugose cushion, tint with purple, margin irregularly denticulate
Hypochile	subconical or helmet-shaped, ca. 2–2.4 × 1.6–2 mm, dorsally compressed, slightly bent outward, the end splits into two conical protrusions	obconical, 3–4 × 2–3 mm, dorsally compressed, slightly bent outward, subacute to obtuse and shortly bifid at apex	subcupular, 4 × 2–3 mm, rounded at end, dorsally compressed, slightly bent outward	subconical, 3 mm tall, 3 mm in diameter, apex rounded

### ﻿Taxonomic treatment

#### ﻿ Gastrochilus heminii

Taxon classificationPlantaeAsparagalesOrchidaceae

M.Liao, B.Xu & Yue H.Cheng sp. nov.

5D856BB6-2D01-5313-A30D-1B910B13202E

urn:lsid:ipni.org:names:77310051-1

Figs 2A, B , 3

##### Type.

China. Sichuan: Wenchuan, coniferous and broadleaf mixed forest, on tree trunk, elev. ca. 2640 m, in flowering and fruiting, 15 March 2022, *Min Liao & Yue-Hong Cheng ZJY143* (holotype CDBI!).

##### Diagnosis.

*Gastrochilusheminii* is morphologically related to *G.affinis* and *G.yei* based on vegetative and floral characteristics such as similar habit, distichous and alternate leaves, epichile surface smooth and glabrous, sepals and petals with purplish-red patches. However, it can be differentiated from *G.affinis* on the basis of flower numbers (1–2 in the former vs. 1–4 in the latter), peduncle length (0.4–0.7 cm in the former vs. 1.5–2.0 cm in the latter) and an additional morphological characteristic: young leaves are densely covered with purple-red spots and old leaves have hardly any purple-red spots in the former (both have purple-red spots in the latter); the reniform epichile is rolled downwards, smooth and glabrous above, and central thickened purple-red mat with irregular folds in the former (subtriangular epichile curves upwards, with 2 thick, brown to purplish-brown median ridges from base to apex in the latter). It differs from *G.yei* by having reniform and lobed epichile (not lobed in the latter), apex of hypochile bilobed and splits into two conical protrusions (not bilobed in the latter), apex of the leaf with 1–2 lobules, lobes setaceous (apex of leaf with a tine in the latter).

**Figure 3.**
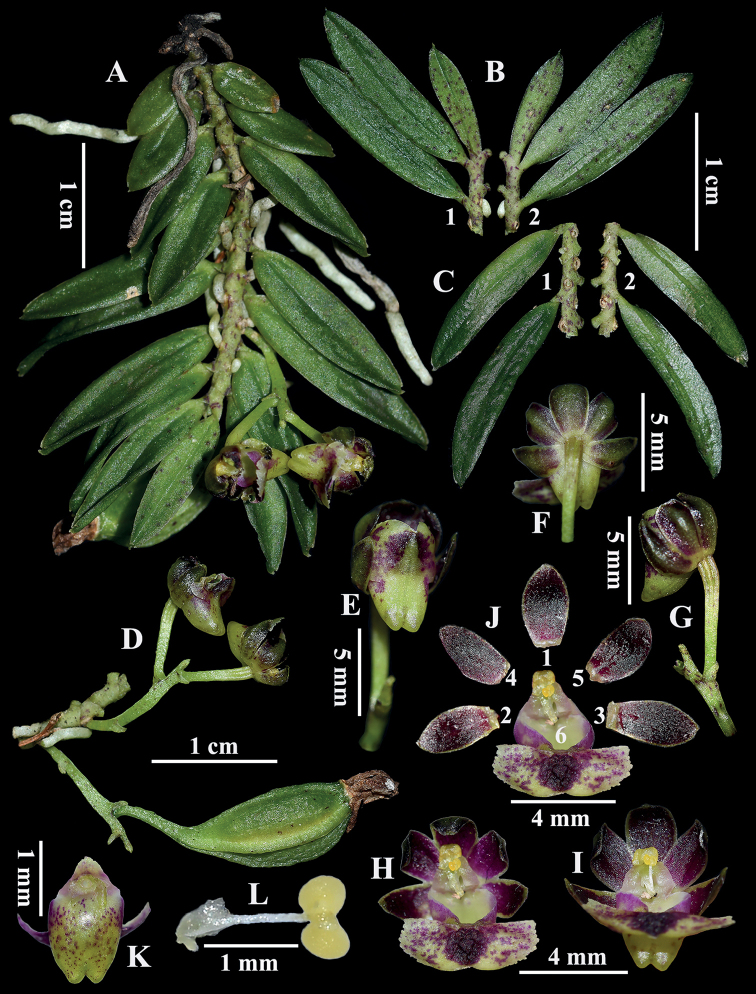
*Gastrochilusheminii***A** flowering plant **B** young leaves (B1: front view; B2: reverse view) **C** old leaves (C1: front view; C2: reverse view) **D** raceme and capsule (side view) **E–I** flowers (different views) **J** anatomy of flower (J1: dorsal sepals; J2 and J3: lateral sepals; J4 and J5: petals; J6: labellum) **K** hypochile (dorsal view) **L** stamens.

##### Description.

Miniature trunk epiphyte. Roots vermiform, 2.0–7.0 cm long and ca. 1.5 mm thick. Stem pendulous, slender, purplish-red spots, 3.0–6.5 cm long and ca. 1.8 mm thick. Leaves alternate, distichous, narrowly oblong or falcate oblong, 0.9–2.3 × 0.3–0.5 cm, apex acute and with 1–2 lobules, lobes setaceous, young leaves with conspicuous purplish-red spots, old leaves with hardly any purplish-red spots. Raceme with 1 or 2 flowers; inflorescence stalk curved upward and thickened, 4.0–9.0 mm long, proximally covered with two sheaths; floral bracts ovate-lanceolate, 0.7–1.0 mm long, apex acute; pedicel and ovary connate, 4.0–5.5 mm long; flowers spreading, ca. 6.0 × 5.0 mm; sepals and petals heterochromatic on both surfaces, outside yellow-green with purplish-red spots, inside purplish-red with yellow-green margin; dorsal sepals and lateral sepals similar and equal in size, elliptic-oblong, ca. 2.4 × 1.5 mm, apex obtuse; petals narrowly oblong, ca. 2.6 × 1.3 mm, apex acute, base narrowed; epichile reniform, yellow-green with purplish-red spots, 4.2–6.5 × 2.0–3.0 mm, margin erose, smooth and glabrous above, central thickened purple-red mat with irregular folds; hypochile subconical, yellow-green with purple-red spots, 2–2.4 × 1.6–2 mm, dorsally compressed, slightly bent outward, the apex splits into two conical protrusions; column cylindrical, ca. 1.0 mm; anther cap subhemispheric, with two chambers, 0.7 × 0.4 mm, hanging from both ends of the stipe; pollinia 2, 0.4 × 0.3 mm, yellow, full and nearly spherical, with a depression in the center; stigma deeply sunken, inverted V-shaped, ca. 0.6 mm long, yellow, apically forked, forked in a subtriangular outline. Capsule shuttle-shaped with six ribs, green with sparse purplish-red spots, ca. 1.1 cm long, inflated to ca. 0.6 cm in the middle, persistent and growing for one year until maturity.

##### Additional specimens examined.

—China. Sichuan: Wenchuan, coniferous and broadleaf mixed forest, on tree trunk, elev. ca. 2640 m, 18 April 2022, *Min Liao & Yue-Hong Cheng ZJY167* (CDBI).

##### Distribution, habitat and phenology.

**Figure 4.**
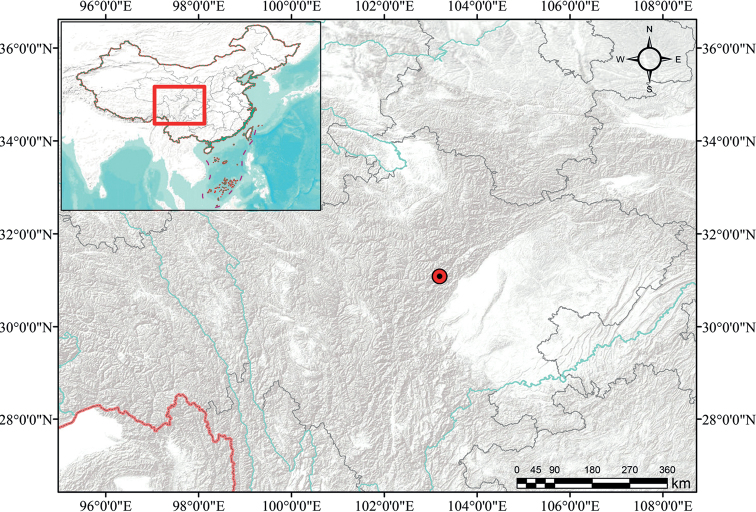
Distribution map of *Gastrochilusheminii*.

The new species is currently known only from Wenchuan County, Sichuan Province, Southwest China (Fig. 4). It is found epiphytic on the trunk of *Tsugachinensis* (Franch.) Pritz. in a subalpine mixed coniferous forest at elevation ca. 2640 m. *Gastrochilusheminii* flowers from March to April.

##### Etymology.

Named in honor of Mr. He-Min Zhang, the advocate of the panda “Release to the Forest”, one of the pioneers of panda research in China, renowned as the “father” of pandas, in recognition of his contribution to the conservation of flora and fauna in the region which was one of the earliest reserves dedicated to preserving the habitat of wild pandas. A Chinese name, *he min peng ju lan* (和民盆距兰), is suggested here.

##### Conservation status.

Three populations with a total of ca. 200 individuals of *Gastrochilusheminii* have been documented during our investigation. However, similar habitat of this new species is widespread in the Wolong Natural Reserve. Therefore, we assessed the conservation status of *Gastrochilusheminii* as DD (Data Deficient) according to the IUCN (2022).

## Supplementary Material

XML Treatment for Gastrochilus heminii

## ﻿Appendix I

**Table A1.** The GenBank accession numbers for DNA sequences used in this study.

Taxa	nrITS	*mat*K	*psb*A-*trn*H	*trn*L-F	*psb*M-*trn*D
*G.acaulis* (Lindl.) Kuntze	KM583455	KM583465	—	—	—
*G.acinacifolius* Z.H.Tsi	KJ733412	KJ733569	KJ733492	KJ733649	MK357216
*G.acutifolius* (Lindl.) Kuntze	MT225573	MW433889	—	—	MK357230
*G.affinis* (King & Pantl.) Schltr.	—	MK357141	—	—	MK357141
*G.alatus* X.H.Jin & S.C.Chen	—	—	—	—	MK357228
*G.bellinus* (Rchb.f.) Kuntze	KY966597	KY966884	MK357164	MK357202	MK357240
*G.bigibbus* (Rchb.f. ex Hook.f.) Kuntze	—	MN124439	MN124439	MN124439	MN124439
*G.calceolaris* (Buch.-Ham. ex Smith) D.Don	MN517123	MK357144	MK357169	MK357204	MK357232
*G.changjiangensis* Q.Liu & M.Z.Huang	MK357124	—	MK357166	—	MK357236
*G.ciliaris* F.Maekawa	—	MK357148	MK357173	—	MK357225
*G.dasypogon* (Lindl.) Kuntze	DQ091685	MK357149	MK357181	MK357197	MK357219
*G.deminutus* J.M.H.Shaw	KY966600	KY966887	—	—	—
*G.distichus* (Lindl.) Kuntze	KJ733414	KJ733571	KJ733494	KJ733651	—
*G.fargesii* (Kraenzl.) Schltr.	KJ733415	KJ733572	—	KJ733652	—
*G.formosanus* (Hayata) Hayata	KJ733416	KJ733573	KJ733495	KJ733653	MK357226
*G.fuscopunctatus* (Hayata) Hayata	MK317970	MK357150	MK357171	MK357192	MK357231
*G.gongshanensis* Z.H.Tsi	—	MN124438	MN124438	MN124438	MN124438
*G.guangtungensis* Z.H.Tsi	KJ733417	KJ733574	KJ733496	KJ733654	KJ733654
*G.heminii* M.Liao, B.Xu & Yue.H.Cheng	ON286752	ON331126	ON331128	ON331130	ON331132
*G.heminii* M.Liao, B.Xu & Yue.H.Cheng	ON286753	ON331127	ON331129	ON331131	ON331133
*G.intermedius* (Griff. ex Lindl.) Kuntze	MK357121	MK357151	MK357172	MK357190	MK357213
*G.japonicus* (Makino) Schltr.	KJ733418	KF545886	KF545866	KF545897	KX871236
*G.linearifolius* Z.H.Tsi & Garay	MK357133	MK357136	MK357187	MK357194	MK357229
*G.linii* Ormerod	—	MK357152	MK357176	MK357198	MK357224
*G.malipoensis* X.H.Jin & S.C.Chen	—	MK357147	MK357177	MK357200	MK357235
*G.minutiflorus* Aver.	—	MK357153	MK357179	—	MK357215
*G.obliquus* (Lindl.) Kuntze	MK357131	MK357137	KJ733498	KJ733656	MK357211
*G.platycalcaratus* (Rolfe) Schltr.	MK357122	—	MK357175	—	MK357222
*G.pseudodistichus* (King & Pantl.) Schltr.	MK357132	—	MK357170	—	MK357221
*G.rantabunensis* C.Chow ex T.P.Lin	—	MK357155	MK357184	MK357193	MK357223
*G.raraensis* Fukuyama	KJ733420	KJ733577	KJ733499	KJ733657	MK357239
*G.sinensis* Z.H.Tsi	OM985813	OK042953	OK172399	OK172401	—
*G.somai* (Hayata) Hayata	MK357128	MN124436	MK357180	MN124436	MK357220
*G.tianbaoensis* Q.Liu & Y.H.Tan	MK357120	MK357157	MK357186	MK357207	MK357214
*G.wolongensis* Jun.Y.Zhang, B.Xu & Yue.H.Cheng	OM985810	OK172400	OK172402	OK172404	OK172403
*G.wolongensis* Jun.Y.Zhang, B.Xu & Yue.H.Cheng	OM985811	OM974209	OM974211	OM974210	—
*G.yunnanensis* Schltr.	MK165469	MK357158	MK357185	—	MK357212
*G.zhenyuanensis* Q.Liu & D.P.Ye	MK357127	MK357146	MK357168	MK357199	MK357237
*Holcoglossumamesianum* (Rchb.f.) Christenson	HQ404389	JF763779	HQ404439	—	—
*Holcoglossumkimballianum* (Rchb.f.) Garay	HQ452901	JF763787	HQ404452	—	—
*Luisiamagniflora* Z.H.Tsi & S.C.Chen	KJ733426	KJ733583	KJ733505	—	KJ733663
*Pomatocalpadiffusum* Breda	AB217576	AB217752	—	—	EF670432
*Pomatocalpaspicatum* Breda	DQ091706	KJ733595	KJ733518	—	KJ733675
*Saccolabiumpusillum* Blume	AB217580	AB217756	—	—	—
